# Laparoscopic Retroperitoneal Repair of a Retrocaval Ureter: A Case Report and Video Demonstration

**DOI:** 10.1155/criu/8373968

**Published:** 2026-08-31

**Authors:** Mohammed Alshehri, Ebtesam Almajed, Khadijah Eid, Nojoud Alamri

**Affiliations:** ^1^ Department of Urology, King Abdullah Bin Abdulaziz University Hospital, Princess Nourah Bint Abdulrahman University, Riyadh, Saudi Arabia, pnu.edu.sa; ^2^ Department of Urology, Prince Sultan Military Medical City, Riyadh, Saudi Arabia, psmmc.med.sa; ^3^ Department of Urology, Indiana University School of Medicine, Indianapolis, Indiana, USA, iusb.edu; ^4^ Toronto Western Hospital, University of Toronto, Toronto, Canada, utoronto.ca

**Keywords:** case report, circumcaval ureter, laparoscopy, retrocaval ureter, retroperitoneoscopy

## Abstract

**Background:**

Retrocaval ureter is an uncommon congenital condition caused by abnormal development of the inferior vena cava. The resulting posterior displacement of the ureter may produce compression, impaired urinary drainage, and hydronephrosis.

**Case Presentation:**

A 35‐year‐old man was referred following the detection of a right‐sided Type I retrocaval ureter during evaluation of right renal colic. Laboratory investigations and physical examination were unremarkable. Computed tomography urography and retrograde pyelography demonstrated the characteristic abnormal ureteral course with associated proximal obstruction. The patient underwent laparoscopic retroperitoneal mobilization, transposition, and reconstruction of the ureter. The operative steps are presented in an accompanying video. Recovery was uncomplicated, and follow‐up imaging demonstrated resolution of the obstruction.

**Conclusion:**

Laparoscopic retroperitoneal reconstruction provides direct access to the affected ureter while avoiding entry into the peritoneal cavity. In appropriately selected patients and experienced hands, it represents an effective minimally invasive option for the treatment of retrocaval ureter.

## 1. Introduction

Retrocaval ureter, also termed circumcaval ureter, is an uncommon congenital condition in which the proximal right ureter follows an abnormal course behind the inferior vena cava before returning to its usual position [1]. Although the abnormality involves the ureter anatomically, its embryological origin lies in atypical development of the inferior vena cava. Persistence of the right subcardinal venous system results in formation of the infrarenal vena cava anterior to the developing ureter, thereby displacing the ureter posteriorly [2].

Retrocaval ureters are generally categorized into two anatomical patterns. Type I is the more common form and is characterized by a low retrocaval crossing with marked medial deviation and a pronounced hook‐ or S‐shaped configuration. This arrangement is frequently associated with proximal ureteral dilatation and hydronephrosis. Type II has a higher crossing point and a less pronounced curvature, with minimal or absent obstruction [3]. The estimated incidence is approximately one per 1000 individuals, with a reported predominance among men and presentation most frequently occurring during the third or fourth decade of life [3].

The clinical manifestations are primarily determined by the degree and duration of urinary obstruction. Patients may present with intermittent right flank pain, recurrent urinary tract infection, hematuria, stone formation, or progressive hydronephrosis [4]. Ultrasonography may identify upper urinary tract dilatation but cannot reliably define the retrocaval relationship. Computed tomography urography is therefore particularly useful because it demonstrates the course of the ureter relative to the inferior vena cava and helps exclude other retroperitoneal causes of obstruction [1].

We describe a right‐sided Type I retrocaval ureter managed using a laparoscopic retroperitoneal approach. To our knowledge, this represents the first reported use of this technique for retrocaval ureter repair in Saudi Arabia. An accompanying operative video demonstrates the principal anatomical landmarks and reconstructive steps. This report was prepared in accordance with the CARE guidelines.

## 2. Case Report

**Figure 1:**
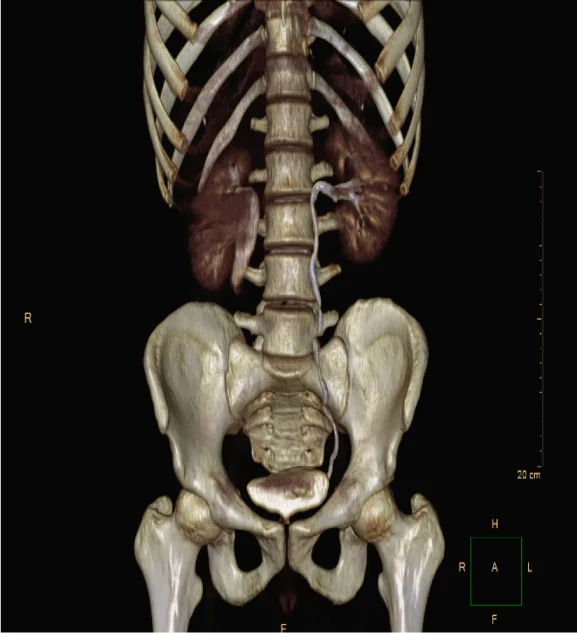
Three‐dimensional reconstructed CT urography demonstrates a right‐sided retrocaval ureter with mild‐to‐moderate proximal hydroureteronephrosis. The proximal ureter shows a posterior and medial deviation consistent with a retrocaval course at the level of the L2 vertebra.

A 35‐year‐old male was referred to the urology clinic with a diagnosis of a right‐sided Type I retrocaval ureter. The patient had initially presented to an outside hospital with right renal colic attributed to an obstructing 5‐mm right ureteric calculus, for which a right double‐J ureteric stent was inserted. Repeat computed tomography urography at our institution demonstrated no residual ureteric calculus. However, it revealed mild‐to‐moderate right proximal hydroureteronephrosis and a posterior and medial course of the ureter behind the inferior vena cava at the level of the L2 vertebra, confirming a Type I retrocaval ureter (Figure 1).

**Figure 2:**
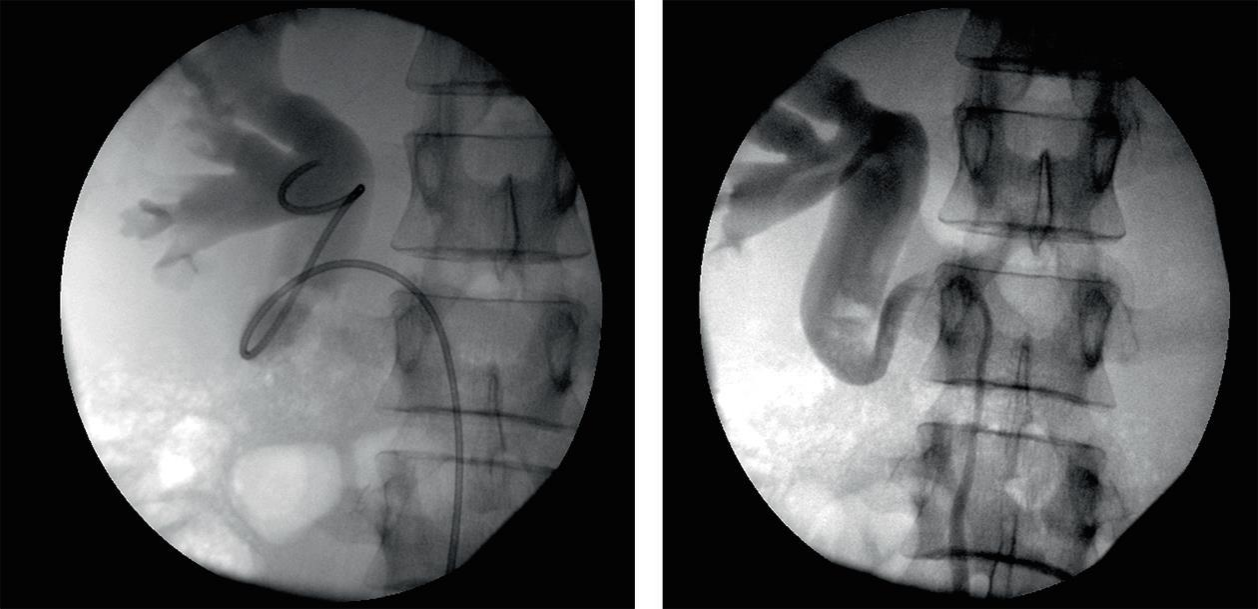
Intraoperative retrograde pyelogram showing right ureteral deviation toward the midline with characteristic S‐shaped dilatation of the proximal ureter, confirming the diagnosis of a Type I retrocaval ureter.

Given the presence of ureteral obstruction and anatomical abnormality, surgical intervention was indicated. The patient was counseled regarding the operative plan, including a laparoscopic retroperitoneal approach, and informed consent was obtained, including consent for intraoperative photography and video recording. The operation was performed on November 28, 2022, under general anesthesia. The patient was initially positioned in the lithotomy position, and a diagnostic cystoscopy was performed using a 21‐Fr cystoscope with a 30° lens, which was unremarkable. A right retrograde pyelogram demonstrated a midline deviation of the right ureter with an S‐shaped dilatation of the proximal ureter, confirming the diagnosis of retrocaval ureter (Figure 2).

**Figure 3:**
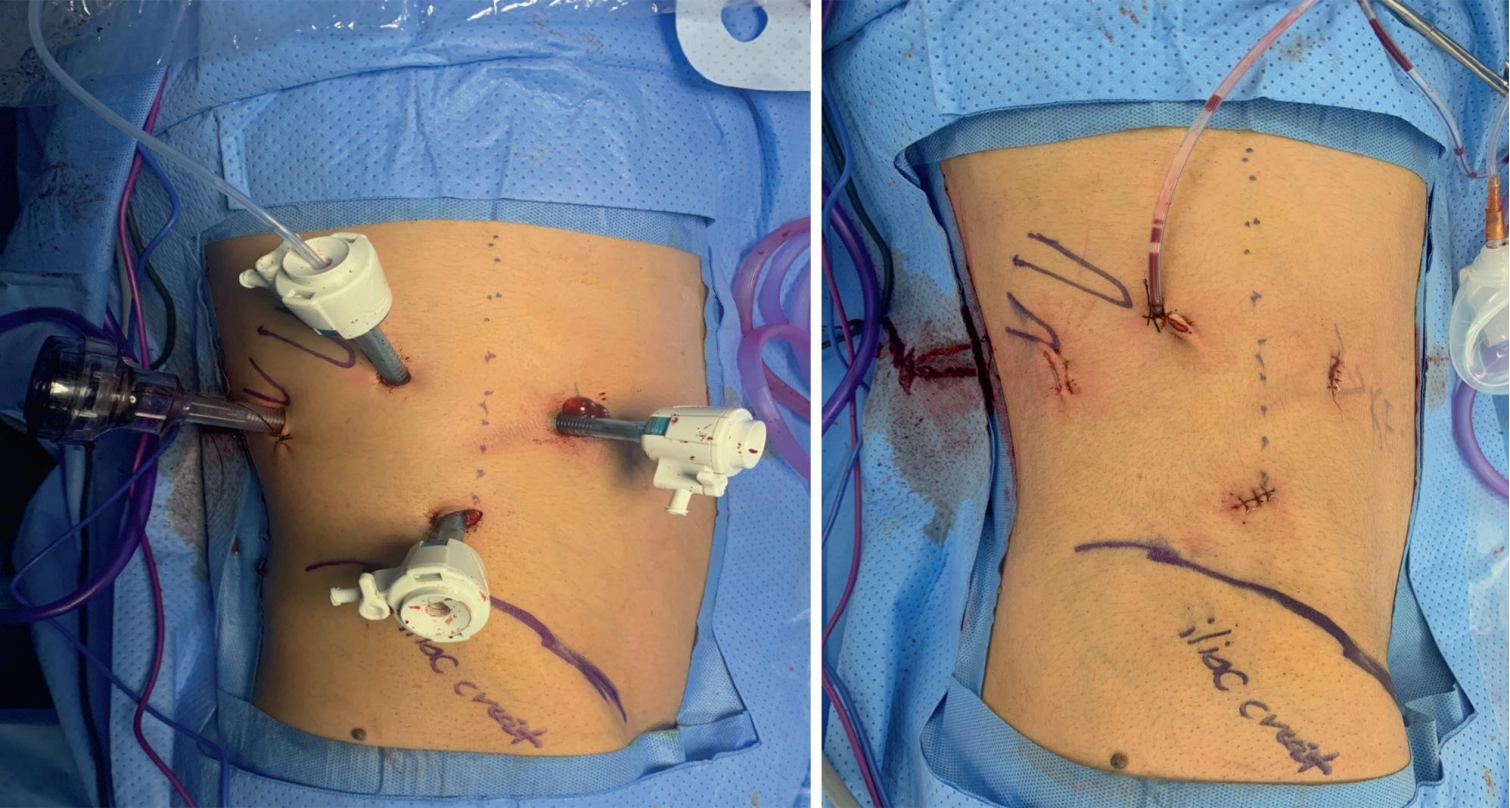
Laparoscopic retroperitoneal port placement demonstrates trocar placement for laparoscopic retroperitoneal repair. A 10‐mm camera port was placed at the tip of the right 12th rib, with three additional 5‐mm working ports positioned along the 11th rib, mid‐axillary line above the iliac crest, and anterior axillary line.

The patient was then repositioned into a right lateral decubitus position with hyperextension to facilitate a laparoscopic retroperitoneal approach. A 1.5‐cm skin incision was made in the posterior axillary line just distal to the tip of the right 12th rib. The lumbodorsal fascia was reached using hemostatic forceps pushed deeply, followed by digital dissection to access and develop the retroperitoneal space. A working space was created using a Spacemaker round balloon, and a 10‐mm port was inserted and fixed in place, with the wound closed to avoid gas air leak. CO_2_ insufflation was established at a pressure of 15 mmHg Three additional 5‐mm ports were placed: one at the tip of the right 11th rib, positioned 3–4 cm anterior to the initial port; a second port along the mid‐axillary line, located 4 cm above the iliac crest and approximately 7 cm inferior to the first port; and a third port along the anterior axillary line, which was particularly useful as a retractor. Port placement for the laparoscopic retroperitoneal approach is illustrated in Figure 3. The pre‐existing double‐J ureteric stent remained in situ during the laparoscopic retroperitoneal dissection. Following trocar placement, a 30° laparoscope was introduced. The psoas muscle was identified and used as an anatomical landmark. Gerota′s fascia was opened, and the perinephric fat was dissected until the dorsal aspect of the renal pelvis and ureter was identified. Nevertheless, the distal ureter could not be confidently identified, and the stent provided little additional visual or instrument‐mediated tactile guidance. Dissection around the pelviureteric junction and proximal ureter was performed, and the inferior pole of the kidney was also identified. The ureter was then followed proximally along the surface of the psoas muscle. The inferior vena cava was gently elevated using dissecting forceps, and the ureter was carefully mobilized and bluntly dissected at the point where it coursed posterior to the vena cava, then located medial to the inferior vena cava. The ureter was transected and transposed to lie lateral to the inferior vena cava in its normal anatomical position. The unhealthy segment and redundant portion of the renal pelvis and proximal ureter, measuring approximately 4 cm, were excised. The ureter was then spatulated for approximately 2 cm along its lateral border. Reconstruction was achieved by performing a pelvis‐to‐ureter anastomosis using 5‐0 Vicryl absorbable sutures, placed in a continuous, watertight, and tension‐free manner over a double‐J ureteric stent. Supporting Information 1: Video S1 highlights key anatomical landmarks and technical steps, illustrating the feasibility and efficiency of the laparoscopic retroperitoneal approach for retrocaval ureter repair. Throughout the procedure, hemostasis was maintained, with minor oozing and bleeding controlled as required. Final inspection confirmed the absence of active bleeding. A 10‐Fr drain was inserted through the trocar site at the tip of the right 11th rib. Closure of the 10‐mm port sites was performed in two layers: fascial closure with Vicryl sutures, followed by skin closure, and the 5‐mm port sites were closed at the skin level only. The total operative time was approximately 150 min, with an estimated blood loss of 50 mL. No intraoperative complications were encountered. The patient was transferred to the recovery unit in stable condition, with an uneventful immediate postoperative course.

Postoperatively, the patient recovered uneventfully and was discharged on Postoperative Day 2. Histopathological examination of the excised ureteric segment demonstrated urothelium with an edematous lamina propria and no evidence of malignancy. The double‐J ureteric stent was removed 6 weeks after surgery. Follow‐up ultrasonography performed at 3 months demonstrated resolution of the hydronephrosis. Diuretic renal scintigraphy performed in 2023 showed preserved bilateral renal perfusion, homogeneous parenchymal uptake, and normal renal function. Split renal function was 42% for the right kidney and 58% for the left kidney. There was no scintigraphic evidence of urinary obstruction, with postfurosemide drainage half‐times of 9 min for the right kidney and 8 min for the left kidney. Minimal residual pelvicalyceal activity was observed on the delayed and postvoid images. The patient remained under documented follow‐up for 8 months after surgery but was subsequently lost to follow‐up. Consequently, updated clinical and radiological outcomes beyond this period were unavailable.

## 3. Discussion

Despite its congenital origin, retrocaval ureter is often diagnosed in adulthood and presents unique diagnostic and therapeutic considerations; its rarity and nonspecific clinical presentation continue to make it a subject of ongoing clinical interest.

Management depends on symptom severity and the presence of obstruction. Asymptomatic patients with minimal hydronephrosis can be managed conservatively with observation and periodic functional studies, given the congenital nature of the condition [3]. However, symptomatic or obstructive cases warrant surgical intervention to relieve the ureteral obstruction and preserve renal function. Open surgery through a transabdominal or retroperitoneal approach was the traditional treatment for many decades [3]. Open ureteral resection and reanastomosis (ureteroureterostomy or pyeloureterostomy). In recent years, minimally invasive surgery has become the preferred approach for retrocaval ureter. Laparoscopic repair and, more recently, robotic‐assisted repair achieve outcomes equivalent to open surgery while markedly reducing perioperative morbidity. Patients undergoing laparoscopy experience significantly less blood loss, less postoperative pain, shorter hospital stays, and superior cosmetic results compared to open surgery [4, 5].

A transperitoneal laparoscopic approach offers a broad operative field and excellent exposure, which facilitates intracorporeal suturing of the ureteric anastomosis. By contrast, a retroperitoneoscopic approach first described by Salomon et al. in 1999 avoids entering the peritoneal cavity and provides more direct access to the posteriorly displaced ureter and IVC [6]. The retroperitoneal route avoids bowel mobilization and may reduce intra‐abdominal complications; however, the limited working space can make delicate dissection and suturing more challenging. The choice between transperitoneal, retroperitoneal, or robotic techniques often depends on the surgeon′s expertise and resource availability, as all approaches appear to have comparable efficacy in experienced hands [3, 4]. In our case, a laparoscopic retroperitoneal ureteroureterostomy was performed with satisfactory outcomes. A recent report by Lema et al. from Northern Tanzania described successful open pyeloureterostomy in two adult patients, underscoring that open reconstruction remains an effective and practical option in resource‐limited settings where laparoscopic or robotic approaches may not be readily available [7].

The principal feature influencing operative management in this case was the patient′s Type I retrocaval anatomy. Computed tomography urography and retrograde pyelography demonstrated marked medial deviation and an S‐shaped proximal ureter with associated hydroureteronephrosis. Intraoperatively, the ureter passed posterior to the inferior vena cava and emerged medially before returning toward the renal pelvis. This configuration was consistent with the obstructive Type I pattern described in previous reports, in which a pronounced retrocaval loop is more frequently associated with proximal dilatation and symptomatic obstruction. The retroperitoneal route was selected because it provided direct access to the posteriorly displaced ureter and inferior vena cava without requiring bowel mobilization. This advantage was particularly relevant in the present case because the abnormal ureteral segment was located immediately behind the vena cava. However, the restricted retroperitoneal working space and close relationship between the ureter and major vessels increased the technical demands of dissection and intracorporeal reconstruction. The procedure was completed in approximately 150 min, with an estimated blood loss of 50 mL, no intraoperative complications, and discharge on Postoperative Day 2. These outcomes are consistent with published minimally invasive series reporting limited blood loss, short hospitalization, and low perioperative morbidity.

Surgical correction of a retrocaval ureter generally carries an excellent prognosis. Relief of the obstruction leads to improvement in hydronephrosis and preservation of renal function in the vast majority of cases [8]. Published series of laparoscopic repairs have reported complete success in restoring ureteric drainage [4]. A thorough literature review by Chen et al. identified 62 patients treated with laparoscopic reconstruction, all with substantial resolution of hydronephrosis on follow‐up imaging [8]. Our patient′s outcome is consistent with these reports; following laparoscopic retroperitoneal repair, he had prompt symptom relief and improved drainage on imaging.

Moreover, postoperative follow‐up should include periodic surveillance imaging, usually through ultrasound, and functional assessment. Prior studies have employed nuclear renography and serial ultrasound at 6–12‐month intervals postrepair to ensure durable success [8]. Long‐term surveillance is important, as late complications, although uncommon, can occur. Reported complications include ureteral stricture at the anastomosis or persistent obstruction if the retrocaval segment was inadequately relocated [3]. Such complications are rare; in a retrospective study, a single patient required reoperation for an anastomotic stenosis [9]. To detect early recurrence of obstruction, we recommend follow‐up imaging and renal function tests at 3–6 months poststent removal, then annually for a couple of years. With appropriate repair and follow‐up, patients can expect excellent long‐term outcomes.

The principal limitation of this report is the absence of long‐term follow‐up. Although ultrasonography and diuretic renal scintigraphy provided objective evidence of satisfactory anatomical and functional outcomes during 8 months of postoperative surveillance, the patient was subsequently lost to follow‐up. Therefore, the durability of the ureteral reconstruction beyond this period cannot be confirmed.

## 4. Conclusion

A retrocaval ureter is a rare congenital anomaly that may lead to clinically significant ureteral obstruction and progressive renal impairment if left untreated. Accurate diagnosis relies on cross‐sectional imaging, and surgical intervention remains the definitive treatment in symptomatic or obstructive cases. This case represents the first reported laparoscopic retroperitoneal repair of a retrocaval ureter in Saudi Arabia and demonstrates the feasibility of this approach in experienced hands. The retroperitoneal route provides direct access to the ureter and inferior vena cava while avoiding intraperitoneal dissection, allowing effective ureteral transposition and reconstruction with favorable perioperative outcomes. Careful patient selection, cautious surgical techniques, and regular postoperative follow‐up are essential to ensure durable relief of obstruction and preservation of renal function.

## Author Contributions

**Mohammed Alshehri:** conceptualization, clinical management, surgical procedure, data acquisition, supervision, and writing—review and editing. **Ebtesam Almajed:** data curation, literature review, writing—original draft, writing—review and editing, and project administration. **Khadijah Eid:** literature review, interpretation of the findings, and writing—review and editing. **Nojoud Alamri:** writing—review and editing.

## Funding

No funding was received for this manuscript.

## Disclosure

All authors read and approved the final version of the manuscript and agreed to be accountable for all aspects of the work. Ebtesam Almajed had full access to all of the data in this study and takes complete responsibility for the integrity of the data and the accuracy of the data analysis.

## Ethics Statement

Formal institutional ethical approval was not required for this single‐patient case report in accordance with the policies of the participating institution. The report was prepared in accordance with applicable institutional ethical standards and the principles of the Declaration of Helsinki.

## Consent

Written informed consent was obtained from the patient for the publication of this case report and any accompanying images.

## Conflicts of Interest

The authors declare no conflicts of interest.

## Supporting Information

Additional supporting information can be found online in the Supporting Information section.

## Supporting information


**Supporting Information 1** Video S1: Laparoscopic retroperitoneal repair of a right‐sided retrocaval ureter. The video demonstrates establishing retroperitoneal access, identifying and mobilizing the right ureter posterior to the inferior vena cava, and confirming a Type I retrocaval course. The ureter is transected proximal to the retrocaval segment, transposed anterior to the inferior vena cava, and reconstructed with a tension‐free ureteroureterostomy over a double‐J stent using intracorporeal suturing.

**Supporting Information 2** CARE checklist.

## Data Availability

The data that support the findings of this study are available in the supporting information of this article.
